# Adjunctive regenerative therapies in hair transplantation: a comprehensive review of platelet-rich plasma, exosomes, and emerging methods

**DOI:** 10.3389/fmed.2026.1820122

**Published:** 2026-07-02

**Authors:** Abdulaziz Balwi, Tamer Koldas

**Affiliations:** 1 Elit Hair Gmbh, Berlin, Germany; 2 Elit Clinic Medical Center, Istanbul, Türkiye

**Keywords:** androgenetic alopecia, extracellular vesicles, hair transplantation, platelet-rich plasma, regenerative dermatology

## Abstract

**Background:**

Adjunctive regenerative therapies are increasingly used to improve outcomes in hair transplantation; however, evidence remains heterogeneously and variably reported.

**Objective:**

To provide a structured, evidence-based narrative review of platelet-rich plasma (PRP), extracellular vesicles/exosomes, and emerging adjunctive therapies in the context of hair transplantation.

**Methods:**

A structured literature search was conducted in PubMed/MEDLINE, Scopus, and Google Scholar databases from database inception to February 2025. English-language studies evaluating regenerative therapies in hair loss and hair transplantation were included. Study selection followed predefined inclusion and exclusion criteria, and evidence was synthesized narratively, with emphasis on study design, sample size, quantitative results, and methodological limitations.

**Results:**

PRP shows moderate to strong evidence in androgenetic alopecia; increases in hair density of approximately 10–30 hairs/cm^2^ have been reported in randomized trials, but evidence specific to transplantation is limited and heterogeneous. Exosome-based approaches show preclinical effects on dermal papillary cell proliferation and angiogenesis, but lack robust clinical validation and standardized characterization. Emerging adjuvant therapies, including low-level laser therapy, microneedling-assisted application, and photoactivated PRP, exhibit variable efficacy with limited transplantation-specific data.

**Conclusion:**

PRP represents the most evidence-supported adjuvant therapy in hair transplantation, while exosome-based and emerging regenerative approaches are under investigation. Standardization of protocols, quantitative outcome reporting, and transplantation-specific endpoints are necessary to improve clinical translation and quality of evidence.

## 1 Introduction

Hair loss represents a significant global health and quality of life issue affecting men and women across diverse age groups and ethnicities. Androgenetic alopecia (AGA) remains the most common cause, with a lifetime prevalence exceeding 50% in men and up to 40% in women, while other conditions such as telogen effluvium, alopecia areata, and scarring alopecia also contribute to the overall disease burden (1, 2). The psychosocial impact of hair loss is significant, including impaired self-esteem, social anxiety, and reduced quality of life, reinforcing the importance of effective and lasting treatment strategies (3–5). In parallel, demand for hair restoration procedures appears to have increased globally over the past decade, reflecting the high prevalence of hair loss and rising patient expectations for durable aesthetic outcomes (6).

Hair transplantation, offering permanent follicular redistribution through techniques such as follicular unit extraction (FUE) and follicular unit transplantation (FUT), has become the most definitive treatment method for advanced hair loss. Advances in instrumentation, graft processing, and implantation techniques have improved survival rates and cosmetic outcomes; however, hair transplantation alone does not alter the underlying pathophysiology of hair loss, including follicular miniaturization, inflammatory signaling, and microvascular disruption (7, 8). Consequently, there has been increased interest in adjunctive therapies that can improve graft survival, accelerate postoperative recovery, and support long-term follicular health (3).

Regenerative medicine-based approaches have emerged as promising adjunctive therapies in hair transplantation, with platelet-rich plasma (PRP) being the most widely adopted method in clinical practice. PRP is an autologous blood-derived product enriched with platelets and biologically active growth factors, including platelet-derived growth factor, vascular endothelial growth factor, transforming growth factor-*β*, and insulin-like growth factor-1 (9). All of these play a role in angiogenesis, cell proliferation, and the follicular cycle (10, 11). Numerous clinical studies and meta-analyses have shown that PRP, when used alone or in addition to hair transplantation, demonstrates improvements in hair density, shaft diameter, and patient satisfaction; however, heterogeneity in preparation protocols and outcome reporting remains a significant limitation (8, 12).

Beyond conventional PRP, there is increasing interest in extracellular vesicles, particularly exosomes, as potential mediators of regenerative effects in hair restoration. Exosomes are nanoscale vesicles released by platelets, mesenchymal stem cells, and other cell types that carry proteins, lipids, and microRNAs involved in intercellular communication and tissue regeneration (13, 14). Preclinical and early clinical studies suggest that exosome-rich preparations may stimulate dermal papilla cell proliferation, enhance angiogenesis, and modulate inflammatory pathways related to hair follicle regeneration (15, 16). However, regulatory uncertainty, limited standardization, and variability in sourcing and characterization currently restrict widespread clinical application.

In parallel, new strategies aimed at enhancing the biological activity of autologous therapies have begun to attract interest among hair transplant practitioners. Common practices for these strategies include photothermal or photoactivated PRP, low-level laser therapy (LLLT), microneedling-assisted application (MAA), and combination regenerative protocols. These approaches aim to optimize growth factor release, exosome production, and cellular metabolism without the use of exogenous or allogeneic products, while maintaining favorable safety and regulatory profiles (17, 18). Despite increasing clinical interest, the evidence base supporting these methods remains fragmented and shows significant differences in study design, outcome measures, and mechanistic validation.

Given the increasing use of regenerative adjuncts in hair transplantation and the lack of a unified clinical guideline, a comprehensive and critical evaluation of the available evidence is necessary. This review aims to synthesize the biological rationale, clinical data, and practical considerations surrounding platelet-rich plasma, exosome-based approaches, and novel regenerative methods used in conjunction with hair transplantation. This review is intended to provide hair transplant practitioners with a balanced and evidence-based framework to support clinical decision-making and future research by clearly distinguishing established evidence from experimental strategies. Accordingly, this review focuses on preoperative and postoperative regenerative adjuncts used in conjunction with surgical hair restoration, emphasizing clinical applicability, safety, and evidence maturity.

## 2 Methods

### 2.1 Literature search strategy

A structured narrative literature search was conducted to identify studies evaluating regenerative adjunctive therapies in hair transplantation and hair loss. Electronic searches were performed in PubMed/MEDLINE, Scopus, and Google Scholar databases from database inception to February 2025. The search was limited to English publications.

Search terms included combinations of the following keywords and Medical Subject Headings (MeSH): hair transplantation, follicular unit extraction, platelet-rich plasma, PRP, exosomes, extracellular vesicles, low-level laser therapy, microneedling, and hair restoration. Boolean operators (“AND,” “OR”) were applied to refine the results. Reference lists of relevant review articles were manually screened to identify additional studies.

### 2.2 Study selection and eligibility criteria

Studies were included if they met one of the following criteria: (i) randomized controlled trials, (ii) prospective or retrospective clinical trials, (iii) systematic reviews/meta-analyses, or (iv) preclinical studies of translational significance related to hair biology. Studies were required to evaluate regenerative or biologically active therapies either as standalone treatments for hair loss or as adjuncts to hair transplantation.

Exclusion criteria included non-peer-reviewed materials, case reports with fewer than three patients, studies not related to scalp hair disorders, and publications in non-English.

### 2.3 Study screening and data extraction

Titles and abstracts were screened for suitability, followed by a full-text review. Data were extracted focusing on study design, sample size, intervention protocols, outcome measures (e.g., hair density, diameter, graft survival), and reported statistical significance, where available. In reported cases, transplantation-specific outcome points were evaluated individually, including graft survival or hair yield, postoperative hair density, hair shaft diameter, shock loss, time to visible regrowth, wound healing parameters, and patient-reported satisfaction or quality-of-life outcomes.

### 2.4 Evidence synthesis and appraisal

Given the heterogeneity in study design and outcome reporting, a quantitative meta-analysis was not feasible. Instead, findings were synthesized narratively, with emphasizes on quantitative clinical outcomes where reported, study size and methodological quality, consistency of findings across studies, limitations, and potential sources of bias. Evidence strength was categorized qualitatively (strong, moderate, limited, preclinical) depending on study type and consistency.

### 2.5 Limitations of the review approach

This review is limited by its narrative design, potential selection bias, and the heterogeneity of the studies included. Although a structured approach was used, formal systematic review guidelines (e.g., PRISMA) were not fully implemented.

## 3 Platelet-rich plasma as an adjunctive in hair transplantation

### 3.1 Biological rationale and mechanisms of action

PRP exerts its regenerative effects primarily through platelet activation, followed by the release of growth factors and extracellular vesicles that influence angiogenesis, cell proliferation, and inflammation. Upon activation, platelets release a wide range of biologically active mediators, including platelet-derived growth factor (PDGF), vascular endothelial growth factor (VEGF), transforming growth factor-*β* (TGF-β), and insulin-like growth factor-1 (IGF-1), which collectively influence angiogenesis, cellular proliferation, extracellular matrix remodeling, and inflammatory modulation (10, 11).

In the context of hair biology, these growth factors play a central role in maintaining follicular homeostasis and promoting the transition to the anagen phase of the hair cycle. VEGF-mediated angiogenesis enhances vascular supply, which is critical for both native follicle function and the survival of transplanted grafts. PDGF and IGF-1 stimulate dermal papilla cell proliferation and inhibit apoptosis, while TGF-*β* participates in the follicular cycle and tissue remodeling (7). In addition to growth factor signaling, PRP has been shown to exhibit immunomodulatory effects, reducing pro-inflammatory cytokine expression and oxidative stress in the scalp microenvironment; this may be particularly important in conditions characterized by subclinical inflammation. Mechanistic models suggest that PRP can promote anagen elongation through growth factor-mediated activation, including Wnt/*β*-catenin, ERK, and Akt signaling (19).

**Figure 1:**
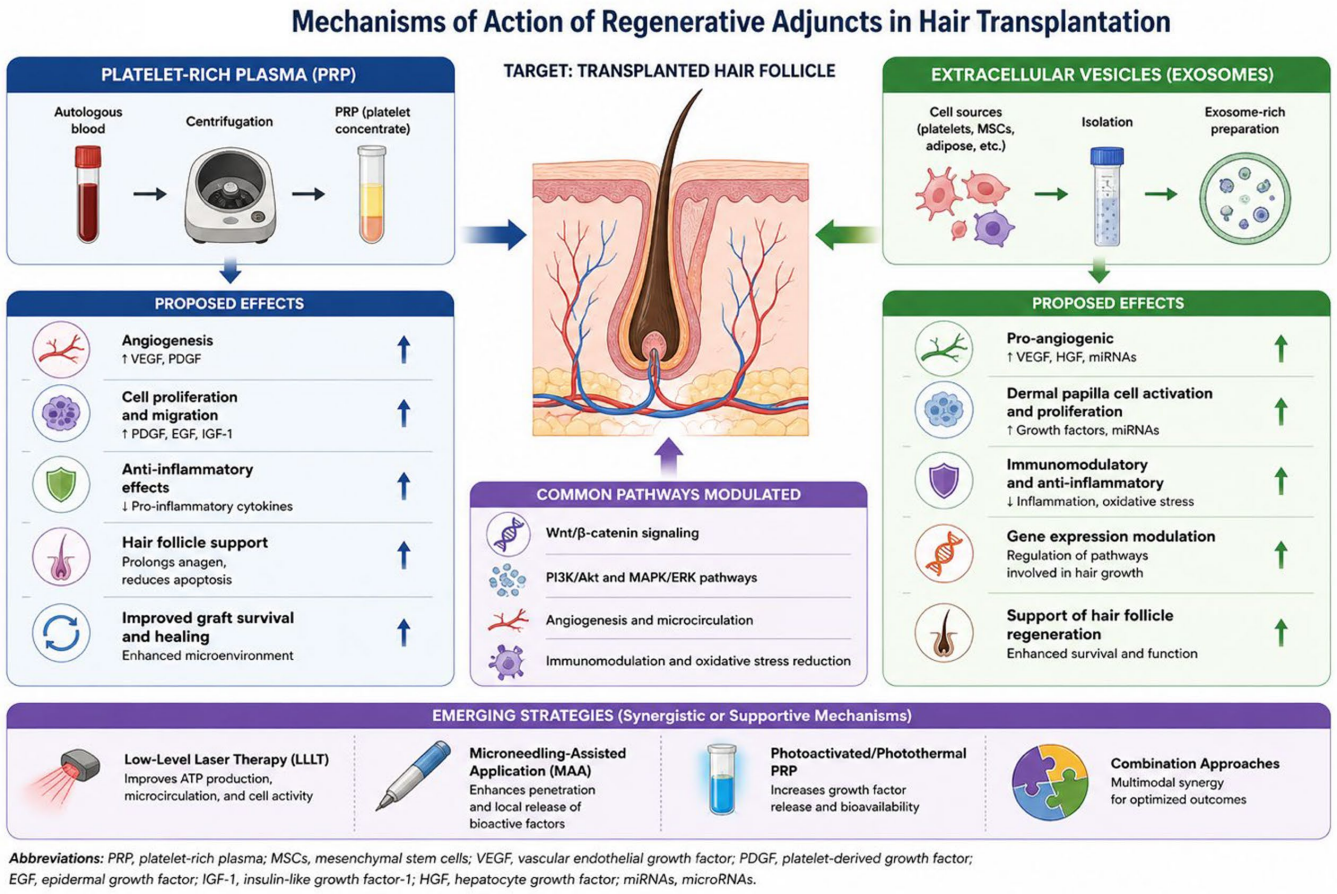
Proposed mechanisms of adjunctive regenerative therapies in hair transplantation. PRP, extracellular vesicles/exosomes, and emerging adjunctive methods are suggested to modulate angiogenesis, inflammatory signaling, and follicular cell pathways in the peri-transplant scalp microenvironment. For extracellular vesicles, a distinction is made between platelet-derived EVs (autologous, produced during PRP activation) and mesenchymal stem/stromal cell (MSC)-derived EVs (cell culture, experimental) as they differ in composition, regulatory status, and clinical evidence base. This scheme summarizes the mechanistic themes discussed in Sections 3–5.

Emerging evidence suggests that extracellular vesicles, including platelet-derived exosomes released during PRP activation, may be a key mediator of these regenerative effects. These vesicles facilitate intercellular communication by delivering microRNAs and proteins involved in angiogenesis, stem cell activation, and inflammatory regulation, thus extending the biological activity of PRP beyond the immediate release of soluble growth factors (10) (see Figure 1).

### 3.2 Clinical evidence for PRP in hair restoration

A growing clinical literature supports the use of PRP in the management of androgenetic alopecia, both as a standalone treatment and in addition to hair transplantation. Quantitative results from randomized controlled trials in androgenetic alopecia show that PRP can increase hair density by approximately 10–30 hairs/cm^2^ compared to baseline or control groups, and statistically significant improvements have been reported in many studies. Improvements in hair shaft diameter and patient-reported outcomes have also been identified (8, 12).

In the context of hair transplantation, however, the evidence is more limited and heterogeneous. PRP has been applied at several stages of the surgical process, including graft handling, recipient site preparation, intraoperative infiltration, and postoperative scalp injections. A prospective randomized study reported improved early postoperative parameters in PRP-treated patients undergoing FUE compared with controls (20). Randomized data also suggest that PRP used as an intraoperative retention solution may increase postoperative hair density compared to standard solutions; however, replication and protocol standardization are needed (21). However, transplantation-specific endpoints such as graft survival rate, hair yield, and regrowth time are reported inconsistently across studies. Experimental data also further suggest that PRP may influence follicular viability during graft preservation, but storage solution and temperature appear to be the main co-determinants (22).

Patient-reported outcomes have also shown positive effects, with improvements observed in satisfaction and quality of life measures after PRP-assisted hair restoration (4, 5). Nevertheless, transplantation-specific outcome points such as graft survival rate, hair yield, regrowth time, shock loss, and long-term durability have been reported inconsistently across studies.

Despite these encouraging findings, the strength of the evidence remains limited due to heterogeneity in PRP preparation protocols, treatment schedules, and outcome assessment methods. Differences in centrifugation techniques, platelet concentration targets, activation strategies, and injection timing contribute to variability between studies, making direct comparisons and combined analyses difficult. Furthermore, many studies involve small sample sizes and short follow-up periods. Therefore, although PRP has consistent biological validity and moderate clinical evidence in androgenetic alopecia, its transplantation-specific efficacy is not yet fully defined and requires more standardized research.

### 3.3 PRP preparation and protocol variability

One of the biggest challenges in interpreting PRP results is the lack of standardization in preparation and application protocols (9). PRP formulations can vary significantly in terms of platelet concentration, leukocyte content, and activation status, each of which can affect biological activity. Leukocyte-rich and leukocyte-poor PRP preparations have been used in hair restoration, but their relative advantages remain unclear due to inconsistent reporting and limited direct comparisons. Protocol diversity remains a significant barrier to interpretation, as demonstrated even in controlled settings comparing PRP injection programs in androgenetic alopecia (23).

Activation methods also vary from endogenous activation via tissue contact to exogenous activation using calcium salts, thrombin, or physical stimulants. These approaches can potentially alter clinical outcomes by affecting the timing and magnitude of growth factor and extracellular vesicle release. Furthermore, the frequency and timing of PRP application (preoperative, intraoperative, or postoperative) relative to hair transplantation are not standardized, further contributing to outcome variability (7). Recent evidence syntheses provide moderate evidence that PRP improves hair density and significant patient outcomes in androgenetic alopecia, while continuing to emphasize the importance of formulation and protocol differences (24, 25). A recent systematic review focusing specifically on the use of PRP as an adjunct to hair transplantation found overall signals of benefit but highlighted the limited number of trials and heterogeneity (26).

### 3.4 Safety considerations and limitations

Platelet-rich plasma (PRP) is generally considered a safe intervention due to its autologous origin and reported side effects are typically limited to transient injection site discomfort, erythema, or edema (7, 11, 25). Serious complications are rare when PRP is prepared and administered under appropriate sterile conditions. However, safety reporting remains inconsistent across clinical trials, and long-term follow-up data following repeated PRP applications are limited (8).

Clinically, it is important to acknowledge that PRP does not reverse advanced follicular miniaturization or replace surgical intervention in appropriately selected patients. Rather, PRP should be considered a biologically active adjunct that, when integrated into a comprehensive treatment strategy, can optimize the scalp microenvironment, support graft survival, and improve overall outcomes (10). Clear patient selection criteria, realistic expectation management, and transparent discussion of evidence limitations are essential to prevent overtreatment or dissatisfaction. Benefits are not observed in all transplantation settings; for example, in FUE-treated cicatricial alopecia, PRP was observed in one study not to significantly affect graft survival, highlighting the dependence on the context of the disease and methodological variability (27).

### 3.5 Practical considerations for hair transplant practitioners

For hair transplant practitioners, the integration of PRP into surgical protocols requires consideration in terms of workflow, cost, and evidence-based benefit. While PRP has been widely adopted in clinical practice, its use should be guided by standardized preparation methods, clearly defined indications, and consistent outcome monitoring (7, 10). Patient-reported outcome measures suggest that PRP, when used appropriately, can positively contribute to postoperative satisfaction, but variability in protocols limits comparability between studies (4).

Importantly, PRP should be differentiated from more experimental regenerative interventions, and patients should be informed about the current level of clinical evidence supporting its adjunctive role (8). Future research priorities include standardized reporting of PRP preparation parameters, comparative studies evaluating formulation and timing strategies, and longer-term follow-up to assess the persistence of benefit (11). A significant limitation frequently encountered in PRP studies is the incomplete characterization of preparation and dosage variables, and evidence syntheses clearly emphasize standardized reporting of isolation protocols to improve reproducibility (28).

Pragmatic protocol frameworks have also been proposed to reduce inter-study variability and facilitate clinical reproducibility (29). An overview of platelet-rich plasma, including its mechanisms of action, maturity of evidence, safety considerations, and research priorities in the context of hair transplantation, is provided in Table 1.

**Table 1:** Summary of platelet-rich plasma as an adjunct in hair transplantation.

Domain	What it is/key points	Practical notes for hair transplant clinics	Evidence strength (hair restoration)	Key references
Definition and components	Autologous platelet concentrate providing growth factors (e.g., PDGF, VEGF, TGF-*β*, IGF-1) and bioactive mediators; may also release platelet-derived extracellular vesicles during activation.	Provide traceable documentation of the preparation phase (spin protocol, target platelet concentration (if applicable), leukocyte content, activation method, injected volume, session schedule).	Moderate-strong (AGA); emerging for specific intraoperative hair transplant endpoints due to heterogeneity.	(7, 10, 11)
Mechanisms related to transplantation	Pro-angiogenic signaling (VEGF), dermal papilla cell support (PDGF/IGF-1), immune modulation, microenvironment optimization; potential contribution from EV/exosome-mediated signaling.	Best framed as an adjunct therapy: optimize recipient bed, reduce inflammation, support early graft environment; do not present it as a replacement for surgery.	Moderate mechanistic plausibility; clinical linkage varies depending on the protocol.	(7, 10)
Clinical efficacy in AGA (standalone)	Meta-analyses/RCT-focused syntheses show improvements in hair density and shaft diameter compared to baseline/placebo in many studies, but effect sizes vary.	Standardize outcome documentation (photographs + trichoscopy), set realistic expectations, schedule care discussions.	Moderate-strong overall, limited by protocol variability.	(8, 12)
Role as an adjunct to hair transplantation	Applied pre-operatively, intra-operatively, and/or post-operatively (scalp injection; sometimes graft exposure/storage); reported benefits include improved early healing and possibly better early yield, but endpoints and methods vary widely between studies.	If used during surgery, write a simple SOP (timing, injection planes/volumes, asepsis). Follow up results prospectively in routine practice to generate publishable data.	Emerging: promising but not uniformly standardized; more high-quality comparative studies are needed.	(7, 8, 11)
Protocol variability (major limitation)	Differences exist in platelet concentration, leukocyte content (LR vs. LP), activation (absent vs. CaCl₂/thrombin/physical), injection depth/interval, number of sessions, and timing according to transplantation.	Clearly report preparation parameters; avoid mixing multiple protocols in a study arm; use consistent follow-up time points (e.g., 3/6/12 months).	Variability is a key reason pooled conclusions are imperfect.	(7, 8, 12)
Safety profile	Generally well tolerated; typical side effects are transient pain, erythema, and edema; serious events are rare with sterile technique and appropriate patient selection.	Use sterile preparation/procedure; document side effects; inform patients that PRP is an adjunct therapy and that outcomes are variable.	Good safety signal, but long-term follow-up reporting is inconsistent.	(8, 11)
Patient-centered outcomes	PRP-associated improvement in patient-reported quality of life has been reported in cases of hair loss; satisfaction appears highest in the early stages of the disease and when expectations are aligned.	Include PROMs (e.g., quality of life/satisfaction scales) in addition to objective measurements; they are useful for both clinical practice and research publications.	Moderate (PROM evidence available; transplant-specific PROM data still limited).	(4, 5)
Key research priorities	Standardization of PRP reporting; head-to-head protocol comparisons; transplantation-specific endpoints (graft survival, growth time, shock loss); longer follow-up; pragmatic and split-scalp designs.	Build a clinical registry system and consider a split-scalp/pragmatic prospective design to produce publishable, scalable evidence.	Field active; highest-value gaps are methodological/standardization.	(7, 8, 10)

## 4 Exosomes in hair transplantation

### 4.1 Biological rationale and definitions

Exosomes are nanoscale extracellular vesicles (typically ~30–150 nm) released by many cell types and play a role in intercellular communication through the transfer of proteins, lipids, mRNA, and microRNAs. In regenerative medicine, exosomes are of interest because they can reproduce some paracrine effects of their parent cells and potentially offer a more standardized, cell-free treatment approach (13, 14). In hair biology, exosome content can influence dermal papilla cell signaling, follicular stem cell activity, angiogenesis, and local inflammatory responses; these processes are directly related to both natural follicle miniaturization and the transplantation microenvironment.

From a practical standpoint, it is essential to distinguish between (i) platelet-derived extracellular vesicles/exosomes that can be produced during PRP activation and (ii) cell-derived exosomes (often discussed in the context of mesenchymal stromal/stem cells) that differ in terms of composition, production, characterization requirements, and regulatory status. This distinction is important because clinical claims, safety assumptions, and generalizability of evidence are not interchangeable between sources (14).

In this review, the term “exosome-based approaches” primarily refers to extracellular vesicles derived from mesenchymal stem/stromal cell sources when discussing products in the investigational phase, while platelet-derived extracellular vesicles are considered as part of PRP biology. These sources should not be interpreted as interchangeable, as they differ in their origins, compositions, production requirements, regulatory status, and levels of clinical evidence.

### 4.2 Preclinical evidence supporting hair growth and follicular support

Experimental studies demonstrate that exosomes derived from mesenchymal stem/stromal cells can promote cellular behaviors related to hair growth, including increased dermal papilla cell proliferation and ex vivo supportive effects on human hair follicles. In a representative mechanistic study, extracellular vesicles derived from mesenchymal stem cells were shown to be associated with growth-promoting effects on dermal cells and follicles, providing a biological rationale for further translational studies (15). Collectively, these data suggest that exosome content (e.g., microRNA profiles and growth-related proteins) may modulate pathways related to anagen induction, perifollicular angiogenesis, and immune balance.

However, preclinical promise does not resolve fundamental translational challenges: exosome preparations vary considerably depending on source cell type, culture conditions, isolation methods, storage conditions, and dosing criteria (particles, protein content, or functional assays). Without standardized characterization, comparing studies or defining dose–response relationships in a clinically meaningful way remains difficult (14).

### 4.3 Clinical status and current limitations

Despite strong preclinical rationale, clinical evidence supporting exosome-based therapies in hair transplantation remains limited. Most current data comes from experimental models or early-stage clinical trials with small sample sizes and heterogeneous methodologies. A recent comprehensive review highlighted significant variability in exosome sources, product characterization, delivery methods, application protocols, and outcome reporting in hair loss studies (16). To date, high-quality randomized controlled trials evaluating exosome therapies, particularly as adjuncts to hair transplantation, are lacking. Reported clinical outcomes remain inconsistent, and key parameters such as dosage, treatment timing, route of administration, and product characterization are not standardized. From a transplantation perspective, the most defensible conceptual role for exosomes is to be seen as an experimental adjunct aimed at optimizing the recipient site microenvironment, rather than replacing surgical follicular redistribution.

Consequently, exosome-based interventions should currently be considered investigational. The clinical use of these treatments should be approached cautiously, and patients should be informed about the limited evidence base, lack of standardized protocols, and regulatory uncertainties surrounding these treatments.

### 4.4 Regulatory and ethical considerations for clinical adoption

The regulatory classification of exosome-based products is complex and depends on the source, degree of manipulation, intended use, and jurisdiction. Unlike standard PRP derived from autologous blood, exosome products (especially those derived from cultured cells) present additional regulatory expectations for manufacturing controls, characterization, efficacy testing, sterility assurance, and batch consistency. The MISEV2018 framework emphasizes minimum reporting standards for extracellular vesicle studies, which are critical for evaluating claims and reproducibility (14). Therefore, in clinical writing and patient communication, it is important to clearly distinguish established autologous procedures from investigational exosome-based interventions and avoid overstating efficacy in the absence of robust controlled trials.

### 4.5 Practical implications and research priorities

Unmet fundamental needs for hair transplant practitioners include: (i) standardized exosome characterization and dosing criteria, (ii) controlled clinical trials with objective outcome points (density/diameter based on trichoscopy, standardized photography, and validated PROMs), and (iii) clear documentation of safety and side effects. Pragmatic study designs feasible in high-volume clinics (e.g., split-scalp comparisons or stepped wedge implementation) may enable a higher-quality evidence generation with minimal workflow disruption. Table 2 outlines the key features and limitations of exosome-based regenerative approaches in hair transplantation.

**Table 2:** Overview of exosome-based approaches in hair transplantation.

Domain	What it is/key points	Practical notes for hair transplant clinics	Evidence strength (hair restoration)	Key references
Core Definition	Exosomes are extracellular vesicles (~30–150 nm) carrying proteins, lipids, mRNA, and microRNAs, and facilitating intercellular communication; they are increasingly being studied as “cell-free” regenerative agents.	Use the term “extracellular vesicles (EVs)” where appropriate, as the term “exosome” is often used loosely in clinical marketing; define the terminology once in the article.	Established biology, early clinical translation.	(13, 14)
Why they matter for hair transplantation	Exosome content can influence dermal papilla signaling, follicular stem cell activation, angiogenesis, and inflammatory modulation; these processes are related to graft handling, early growth, and the recipient site microenvironment.	Frame as an adjunct to optimize recipient bed and perioperative environment, not as a replacement for surgical redistribution.	Preclinical support, limited clinical outcomes in transplant patients.	(14, 15)
Major sources discussed in practice	(i) Platelet-derived EVs/exosomes (typically in/alongside PRP) vs. (ii) cell-derived EVs (typically MSC-derived) vs. (iii) mixed/poorly defined “exosome products.” These are not interchangeable.	Require precise reporting of the source and manufacturing context; avoid confusing autologous point-of-care products with cultured cell derivatives.	Source dependent; clinical maturity varies widely.	(13, 14)
Platelet-derived EVs (PRP-linked)	EVs released during platelet activation may contribute to the regenerative signaling of PRP (angiogenic, immunomodulatory, growth-promoting signals).	If PRP-associated EVs are mentioned, keep claims conservative and base them on the PRP literature; avoid equating PRP-EVs with commercial exosome products.	Indirect clinical significance (via PRP evidence); few transplantation trials have quantified EVs directly.	(10, 14)
MSC-derived EVs (mechanistic hair data)	Preclinical and ex vivo studies suggest that MSC-derived EVs can support dermal papilla cell proliferation and human follicle biology (e.g., hair elongation signaling), supporting translational interest.	Preclinical plausibility ≠ clinical efficacy; if used clinically, documentation should include product characterization and inform the patient about the investigational status.	Preclinical to early clinical.	(13, 15)
Delivery routes (reported/used)	In the hair field, application is generally discussed as intradermal injection, microneedling-assisted application, topical-assisted approaches, or combined protocols.	Standardize the route of administration, injection plane, volume, number of sessions, and timing according to transplantation; avoid changing multiple variables simultaneously in a study.	Early; heterogeneous reporting limits comparisons.	(16)
Characterization and reporting standards	EV/exosome studies require standardized reporting: isolation method, particle size/number, protein markers, purity, storage, and functional testing; MISEV2018 provides minimum information expectations.	Clinics planning research should collaborate with laboratories capable of performing MISEV-compliant characterization (NTA, marker panels, contaminants, stability).	A critical facilitator of reliable clinical evidence.	(14)
Safety considerations	Safety depends on the source and production: autologous PRP-linked EVs differ fundamentally from allogeneic/cultured cell-derived products; contamination and batch variability are significant concerns beyond strict quality control.	Implement adverse event tracking; avoid exaggerated safety claims; ensure sterility assurance and traceability if any EV product is used in research settings.	Lack of safety evidence in hair indications; standardized reporting is needed.	(14, 16)
Regulatory and ethical considerations	Exosome products generally face higher regulatory scrutiny than point-of-care PRP due to manipulation, sourcing, and production requirements; terminology used in marketing can precede evidence.	Patient consent should clearly state the research status; avoid implying regulatory approval for hair loss unless explicitly documented.	Regulatory pathway complex and evolving.	(14, 16)
Clinical evidence status in hair loss	Published clinical evidence is limited and heterogeneous; comprehensive reviews highlight the investigative nature of many applications, emphasizing variability in products, methods of administration, and outcome reporting.	The best short-term approach is to participate in controlled trials or structured registries with objective outcome points and transparent reporting.	Early/emerging.	(16)
High-value research priorities	Standardized dosing criteria (particles vs. protein vs. potency), controlled clinical designs (split-scalp, pragmatic cohort), objective endpoints (trichoscopy, standardized photographs), patient-reported outcome measures (PROMs), and long-term follow-up are key considerations.	High-volume clinics can lead pragmatic/split-scalp studies and contribute to real-world evidence if protocols are standardized and outcomes are systematically recorded.	The field needs methodological improvements rather than a new wave of excitement.	(14–16)

## 5 Emerging adjunctive approaches in hair transplantation

### 5.1 Rationale for emerging adjunctive approaches

**Figure 2:**
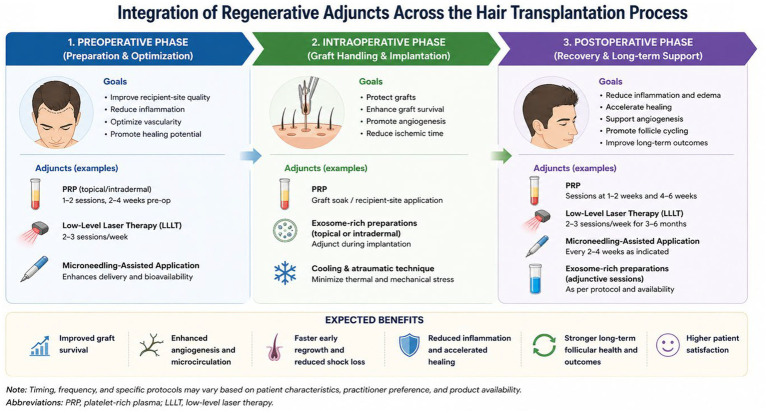
Timing of adjunctive regenerative therapies relative to hair transplantation. A timeline showing common pre- and post-operative periods in which adjunctive approaches can be applied: pre-operative preparation (days to weeks before surgery), intraoperative graft and recipient site manipulation (on the day of surgery), early post-operative recovery (days 1–14), and maintenance phase (1–12 + months). Protocols vary between studies and practices, and maturity of evidence varies by method. Standardized outcome assessment at baseline and follow-up time points (3, 6, and 12 months) is recommended for clinical research and quality monitoring.

Despite advances in surgical techniques and the use of adjunctive PRP, additional peri-transplantation strategies have been explored to optimize the scalp microenvironment and biological signaling related to follicular healing. These approaches generally aim to modulate angiogenesis, inflammation, cellular metabolism, and growth factor bioavailability and are positioned as adjunctive rather than replacement methods for surgical redistribution (30) (see Figure 2).

### 5.2 Photothermal and photoactivated platelet-rich plasma

Photothermal or photoactivated PRP refers to PRP preparations that have been exposed to controlled light or thermal energy stimulation prior to clinical application in order to modify platelet activation, growth factor release kinetics, and downstream biological signaling. Photothermal or photoactivated PRP techniques apply controlled energy to PRP preparations prior to application. Experimental studies show that photoactivation can affect the kinetics of growth factor release and related signaling, potentially impacting the biological activity of PRP compared to nonactivated preparations (18). Early clinical trials in dermatological/aesthetic settings report feasibility and acceptable tolerance, but evidence specific to hair transplantation remains limited, and protocol heterogeneity hinders standardized recommendations (17).

### 5.3 Low-level laser therapy

Low-level light/laser therapy (LLLT) has been evaluated in androgenetic alopecia and is generally presumed to support follicular activity through its effects on cellular metabolism and perifollicular microcirculation. Mechanistically, LLLT/LED therapy is thought to act via photobiomodulation, specifically through mitochondrial chromophore activation, increased ATP production, modulation of reactive oxygen species, and downstream red light-mediated dermal-epidermal signaling pathways that may influence follicular cycle and perifollicular repair responses (31). Evidence syntheses and randomized placebo-controlled trials support LLLT as an effective, non-invasive option for hair growth in AGA, although results vary between devices and protocols (32, 33). In the hair transplant setting, LLLT has primarily been investigated as a postoperative adjunct to enhance healing; however, transplant-specific evidence is limited, and at least one study found no significant benefit after a single session, highlighting uncertainty regarding optimal dosing and clinical effect (34).

A recently published systematic review and meta-analysis supports the idea that low-level laser/LED therapy is an effective option for increasing hair density in androgenetic alopecia, while also highlighting the need for better standardization of transplantation-specific protocols (35).

### 5.4 Microneedling-assisted application

Microneedling has been studied as an adjunct method in AGA and may enhance transdermal delivery of topical agents while simultaneously activating wound healing pathways. A randomized, evaluative-blind study reported that microneedling plus topical minoxidil yielded superior results compared to minoxidil alone in men with AGA, supporting the biological possibility for combination strategies (36). In practice, microneedling is also combined with PRP; however, published evidence is heterogeneous, and protocol differences (needle depth, number of sessions, timing relative to transplantation, and concomitant treatments) limit definitive results (37).

### 5.5 Combined regenerative protocols

Combined protocols aiming to target multiple pathways simultaneously (e.g., microneedling with PRP, LLLT with PRP, or photoactivated PRP) are increasingly reported in practice. However, many existing studies are small, non-randomized, or involve multiple concomitant interventions; this also limits the attribution of effects to individual components and reduces comparability between cohorts (30, 37). Photothermal conditioning has been proposed as an approach to enhance PRP biological activity in hair regeneration, but clinical standardization and evaluation specific to transplantation are still limited (9).

### 5.6 Clinical implications and research priorities

From a clinical perspective, emerging adjunctive methods can be integrated as supportive measures, but their use should be guided by evidence strength, safety, and transparent patient counseling. While PRP remains the best-supported regenerative adjunctive therapy, photoactivated PRP should be evaluated as supportive or experimental until higher-quality transplantation-specific studies are conducted that include transplantation-specific LLLT protocols, microneedling-assisted application and combination regimens, standardized outcome points, and longer follow-up periods (32, 34).

### 5.7 Clinical workflow integration and implementation considerations

In routine clinical practice, adjunctive regenerative therapies are generally integrated into hair transplantation protocols within a structured perioperative framework, rather than being applied as isolated interventions. Because transplantation-specific studies remain limited and heterogeneous, evidence from androgenetic alopecia studies also provides mechanistic and clinical context for adjunctive use in surgical hair restoration (23, 28).

In the preoperative period, patient selection typically reflects the degree of miniaturization, donor-recipient balance, and expectations regarding postoperative recovery. In selected candidates, preconditioning strategies may include PRP sessions or low-level laser/LED therapy (LLLT) aimed at optimizing the scalp microenvironment (35). During surgery, PRP can be administered to the recipient site via infiltration and/or graft exposure strategies (e.g., limited wetting or manual intervention approaches) depending on the workflow and surgeon’s preference; transplantation-focused studies have evaluated intraoperative PRP during FUE and PRP as a retainer solution (20, 21).

In the early postoperative recovery period, supportive interventions are generally applied incrementally. PRP injections can be administered in the weeks following transplantation as a supportive measure for wound healing and early follicular recovery, while LLLT protocols can be initiated postoperatively depending on device availability and clinical practice models; however, transplantation-specific evidence remains limited, and at least one study showed no significant benefit after a single LLLT session, highlighting uncertainty regarding optimal dosing (34). Microneedling is generally avoided immediately following surgery, but may be incorporated later, particularly in non-transplanted or thinning areas, to support wound healing pathways and enhance the delivery of topical/biological agents; supportive evidence exists in androgenetic alopecia, but further evaluation of transplant-specific protocols is needed (36).

Importantly, real-world integration highlights the need for standardized documentation of treatment timing, preparation parameters, and outcome assessment. Consistent reporting of objective measurements (e.g., trichoscopy-based density/diameter), standardized photography, and patient-reported outcomes is necessary to translate clinical experience into reproducible evidence and address the protocol heterogeneity observed in PRP studies (23, 28). To clarify the relative maturity of evidence across assisted regenerative approaches, current methods are summarized in Table 3, considering their level of evidence, quantitative results (where available), current clinical role, and key limitations.

**Table 3:** Evidential strength and clinical applicability of regenerative adjuvants in hair transplantation.

Modality	Evidence level	Quantitative outcomes (where available)	Clinical role	Key limitations
PRP (AGA)	Moderate-strong	+10–30 hair/cm^2^ increase; improvement in diameter	Adjunctive/supportive	Protocol variability
PRP (Transplant)	Emerging	Early postoperative density improvement reported in some studies transplantation-specific endpoints include graft survival/hair yield, hair density, hair shaft diameter, shock loss, time to visible regrowth, and wound healing parameters, but reporting remains inconsistent.	Adjunctive	Limited standardized endpoints
Exosomes (MSC-derived)	Preclinical-early clinical	Increased dermal papilla proliferation (experimental)	Investigational	Lack of high-quality RCTs
LLLT	Moderate (AGA)	Modest density improvement compared to placebo	Optional adjunct	Device variability
Microneedling	Moderate (AGA)	Improved results with minoxidil combination	Adjunct (non-transplant areas)	Protocol inconsistency
Photoactivated PRP	Early	Limited pilot data	Experimental	Lack of standardization

## 6 Conclusions and future perspectives

Hair transplantation remains the most definitive treatment for advanced hair loss; however, surgical redistribution alone does not alter the underlying biological environment affecting follicular survival, inflammation, and long-term miniaturization. Therefore, adjunctive regenerative therapies have emerged as supportive strategies aimed at optimizing the post-transplant scalp environment and enhancing postoperative recovery.

Among current methods, platelet-rich plasma (PRP) represents the most established adjunctive regenerative intervention. While moderate to strong evidence supports its role in androgenetic alopecia, transplant-specific applications show promising but heterogeneous results; these results are largely due to variability in preparation protocols and outcome reporting. Newer techniques, including exosome-based approaches and photoactive PRP, low-level laser therapy, microneedling-assisted application, and combination protocols, remain biologically plausible but are supported by early-stage or inconsistent clinical data. Currently, these strategies should be considered as supportive or investigational rather than standard care.

The future of regenerative adjunctive therapies in hair transplantation depends more on methodological improvement than on the introduction of new technologies. Standardized intervention reporting, clearly defined transplantation-specific endpoints, consistent trichoscopic and photographic documentation, and the inclusion of verified patient-reported outcomes are essential. Pragmatic prospective designs, including split scalp and stepped wedge approaches, offer viable avenues for high-volume clinics to produce reproducible, real-world evidence.

In summary, adjunctive regenerative therapies can enhance surgical hair restoration when integrated into an evidence-based framework and implemented alongside transparent patient counseling. A continued emphasis on protocol standardization and rigorous scientific evaluation will determine which approaches ultimately achieve lasting clinical significance in hair transplantation.
